# Editorial: Women in Psychiatry 2021: Psychopharmacology

**DOI:** 10.3389/fpsyt.2022.905084

**Published:** 2022-05-05

**Authors:** Reem Kais Jan, Alfreda Stadlin, Rosana Camarini

**Affiliations:** ^1^College of Medicine, Mohammed Bin Rashid University of Medicine and Health Sciences, Dubai, United Arab Emirates; ^2^College of Medicine, Ajman University, Ajman, United Arab Emirates; ^3^Department of Pharmacology, Institute of Biomedical Sciences, University of São Paulo, São Paulo, Brazil

**Keywords:** women, polypharmacy, methylphenidate, ^1^H NMR—spectroscopy, Neurexan, treatment-resistant depression (TRD), cannabis, mindfulness

Women remain underrepresented in science. Gender-related differences in productivity are impacted by inequalities in a plethora of factors—career lengths, dropout rates, network of collaborations and funding opportunities among others (1, 2). This Research Topic was part of the inaugural Frontiers in Psychiatry “*Women in Psychiatry*” series of article collections. Given that <30% of researchers are women, this Research Topic aimed to promote the work of women scientists in the field of psychiatry, and in particular the subfield of psychopharmacology. It presents a case report of psychostimulants as treatment for attention deficit hyperactivity disorder (ADHD) and comorbid obsessive-compulsive disorder (OCD), two reviews: 1- about synergism between mindfulness and psychedelics in depressive and anxiety disorders; 2- on the use of proton nuclear magnetic resonance spectroscopy (^1^H NMR) in psychedelic research and five original research articles, across a broad range of topics relating to psychopharmacology by women researchers from seven different countries. The five original articles discuss 1- use of polypharmacy and inappropriate prescription in adults with intellectual disabilities; 2- effects of medicinal use of cannabis on anxiety and depression; 3- the prevalence of cannabis use disorder in recreational vs. medical use; 4- effects of Neurexan on susceptibility to distraction; 5- parameters associated with side effects of antidepressants in patients with treatment-resistant depression.

Lonchampt et al. in “*Prevalence of Polypharmacy and Inappropriate Medication in Adults With Intellectual Disabilities in a Hospital Setting in Switzerland*” evaluated the prevalence of potentially inappropriate medications (PIM) and polypharmacy in adults with intellectual disability in a 10-month prospective observational study. Data were collected weekly and reflected an average of ~9 drugs being prescribed per week; most commonly antipsychotics, followed by benzodiazepines and laxatives. All patients were polymedicated with five or more drugs simultaneously. The authors discuss the high prevalence of polypharmacy and inappropriate prescribing in adults with intellectual disability and make a call for the need for specific prescribing and deprescribing guidelines in this population.

In the “*Case Report: Treatment of a Comorbid Attention Deficit Hyperactivity Disorder and Obsessive–Compulsive Disorder With Psychostimulants*,” Dogan-Sander and Strauß reported the second known case of treating adult comorbid OCD and ADHD with extended-release methylphenidate. Treatment of comorbid ADHD and OCD is challenging and the use of psychostimulants in OCD has not been properly established. The patient reported in the case study experienced improvement in both ADHD and OCD-specific symptoms after treatment with extended-release methylphenidate. The authors recommended that the efficacy of methylphenidate as part of the treatment of comorbid OCD and ADHD should be further evaluated by the means of randomized clinical trials.

In a review article, Eleftheriou and Thomas investigated the potential synergistic effects of mindfulness training and psychedelic-assisted therapy in treating depressive and anxiety disorders. The authors discussed the underlying mechanisms of both methods, that include psychological, behavioral and neuromodulatory effects, and reviewed three recent studies that utilized the combination of the two methods, which provided evidence of synergistic effects. Mindfulness potentiated drug-induced ego-dissolution, while psychedelics enhanced meditation intensity.

Vilca-Melendez et al. performed a systematic review to explore the current use, benefits, limitations, and future potential of ^1^H NMR as a metabonomic tool. They exposed a gap in the current literature whereby ^1^H NMR has been mainly used for characterization of psychedelic substances but not for metabolic profiling of biofluids, despite being comparable to mass spectrometry in efficacy. The authors highlighted several potential future directions for the use of NMR in psychedelic research.

The investigational medicinal product Neurexan (Nx4) which is composed of three herbal extracts: Avena sativa, Coffea arabica, Passiflora incarnata, and the mineral salt Zincum isovalerianicum, has been shown to reduce stress-related parameters. Mayer et al. set out to investigate the effects of a single dose of Nx4 vs. placebo on susceptibility to distraction in 39 participants who performed the attention modulation by salience task (AMST), extended by emotional distractors. The authors performed a *post-hoc* reanalysis of data from the NEURIM study and measured reaction time (RT) and event-related potential (ERP) components for processing auditory information while being presented with visual task-irrelevant distracting cues of high or low salience or negative or positive valence. In comparison to placebo, Nx4 was found to reduce susceptibility to distraction by emotional distractors, supported by reduced RT and N2 and N3 ERP amplitudes when presented with positive valence distractors. Typically, N2 and N3 ERP amplitudes and RTs are increased when presented with high salient distractors. Hence, both behavioral and electroencephalography parameters suggested that Nx4 effectively inhibited emotional distraction, showing positive effects on attention and consequently stress reduction.

Treatment-resistant depression is a difficult to treat, disabling condition that is diagnosed after failure to achieve a therapeutic response with two antidepressant drugs. Side effects are one of the main culprits in early termination of treatment and may result in “pseudo-resistance.” Hence, Levy et al. set out to investigate the role of clinical, socio-demographic and environmental factors in the contribution of side effect occurrence to treatment-resistant depression. The study was a cross-sectional, observational, multicenter study of 108 patients with treatment-resistant depression, from the French network of Expert Centers, and investigated the occurrence and profile of side effects of antidepressant drugs. The authors found that the occurrence of side effects was influenced by several demographic factors such as age and sex, and they showed a positive correlation with the intensity of depressive symptoms and history of childhood trauma. On the other hand, side effects were negatively correlated with self-esteem and overall functioning. The authors concluded that if factors influencing medication side effects were known, they could be targeted to allow for continuation of antidepressant treatment which may increase the chances of therapeutic success in this population.

Two original research articles on medicinal cannabis were published in this Research Topic. The first article was an observational study whereby Martin et al. used an online survey to compare general health between people with anxiety and/or depression who used medicinal cannabis (*n* = 368) and those who did not (controls; *n* = 170). The authors found that medicinal cannabis users experienced reduced anxiety and depressive symptoms, better sleep and quality of life, and less pain than non-using controls. However, these results would require replication, validation and optimization of pharmaceutical aspects by future randomized, placebo-controlled trials. The second article compared the prevalence and severity of cannabis use disorder (CUD) between people who used cannabis exclusively for recreational purposes and those who combined recreational and medicinal use. In this study, Rubin-Kahana et al. employed data from the National Epidemiological Survey on Alcohol and Related Conditions III, a US national interview of 36,309 adults aged 18 years or older conducted in 2012–2013. It was found that users who combined recreational and medicinal cannabis use had a higher prevalence of CUD than those who used it for recreational purposes only. Certain characteristics rendered users who combined recreational and medicinal cannabis more likely to develop CUD, including younger age, male gender, being non-white, living in the Midwest of the US, comorbid mental illness, and consumption of greater amounts of cannabis. The authors put forth some public health recommendations regarding the appropriateness of prescribing medicinal cannabis to patients who already use it recreationally, and the importance of assessing risks vs. benefits of cannabis treatment.

## Author Contributions

RJ wrote the editorial. AS and RC contributed to the review of the editorial. All editors edited the Research Topic.

## Conflict of Interest

The authors declare that the research was conducted in the absence of any commercial or financial relationships that could be construed as a potential conflict of interest.

## Publisher's Note

All claims expressed in this article are solely those of the authors and do not necessarily represent those of their affiliated organizations, or those of the publisher, the editors and the reviewers. Any product that may be evaluated in this article, or claim that may be made by its manufacturer, is not guaranteed or endorsed by the publisher.

## References

[1] Astegiano J Sebastián-González E Castanho CT. Unravelling the gender productivity gap in science: a meta-analytical review. R Soc Open Sci. (2019) 6:181566. 10.1098/rsos.181566 31312468 PMC6599789

[2] Huang J Gates AJ Sinatra R Barabási AL. Historical comparison of gender inequality in scientific careers across countries and disciplines. Proc Natl Acad Sci USA. (2020) 117:4609–16. 10.1073/pnas.1914221117 32071248 PMC7060730

